# Serum proteins for monitoring and predicting visual function in patients with recent optic neuritis

**DOI:** 10.1038/s41598-023-32748-5

**Published:** 2023-04-05

**Authors:** Hyo Jae Kim, Eun-Jae Lee, Sang-Yeob Kim, Hyunjin Kim, Keon-Woo Kim, Seungmi Kim, Hyunji Kim, Dayoung Seo, Byung Joo Lee, Hyun Taek Lim, Kwang-Kuk Kim, Young-Min Lim

**Affiliations:** 1 Department of Neurology, Asan Medical Center, University of Ulsan College of Medicine, Seoul, South Korea; 2 Department of Medicine, Asan Medical Institute of Convergence Science and Technology, University of Ulsan College of Medicine, Seoul, South Korea; 3 Department of Convergence Medicine, Asan Medical Center, University of Ulsan College of Medicine, Seoul, South Korea; 4 Department of Ophthalmology, Asan Medical Center, University of Ulsan College of Medicine, Seoul, South Korea; 5 Translational Biomedical Research Group, Asan Institute for Life Science, Asan Meidcal Center, Seoul, South Korea

**Keywords:** Immunology, Autoimmunity, Neuroimmunology

## Abstract

It is unclear whether serum proteins can serve as biomarkers to reflect pathological changes and predict recovery in inflammation of optic nerve. We evaluated whether serum proteins could monitor and prognosticate optic neuritis (ON). We prospectively recruited consecutive patients with recent ON, classified as ON with anti-aquaporin-4 antibody (AQP4-ON), ON with anti-myelin oligodendrocyte glycoprotein antibody (MOG-ON), and double-seronegative ON (DSN-ON). Using ultrasensitive single-molecule array assays, we measured serum neurofilament light chain and glial fibrillary acidic protein (GFAP), and brain-derived neurotrophic factor (BDNF). We analyzed the markers according to disease group, state, severity, and prognosis. We enrolled 60 patients with recent ON (15 AQP4-ON; 14 MOG-ON; 31 DSN-ON). At baseline, AQP4-ON group had significantly higher serum GFAP levels than did other groups. In AQP4-ON group, serum GFAP levels were significantly higher in the attack state than in the remission state and correlated with poor visual acuity. As a prognostic indicator, serum BDNF levels were positively correlated with follow-up visual function in the AQP4-ON group (*r* = 0.726, *p* = 0.027). Serum GFAP reflected disease status and severity, while serum BDNF was identified as a prognostic biomarker in AQP4-ON. Serum biomarkers are potentially helpful for patients with ON, particularly those with AQP4-ON.

## Introduction

Optic neuritis (ON) is an inflammatory disorder of the optic nerve associated with immune-mediated chronic demyelinating diseases of the central nervous system (CNS), such as multiple sclerosis (MS), neuromyelitis optica spectrum disorder (NMOSD), and myelin oligodendrocyte glycoprotein (MOG) antibody-associated disorder^1–5^. Although these disorders manifest similar ON symptoms, their underlying pathogenic mechanisms are disease-dependent^6,7^.

ON in these conditions tends to relapse and can result in degeneration of the optic nerve^8,9^. Because the visual function is essential for daily activities, its impairment negatively affects quality of life^10^. Serum biomarkers to monitor disease severity and predict functional outcomes would be helpful in clinical management of ON. With the recent development of ultrasensitive single-molecule array (Simoa) analysis^11^, several clinically relevant serum proteins have been identified for multiple neurological disorders^12–17^. However, because the optic nerve constitutes only a small proportion of CNS tissue, it is unclear whether serum protein levels can adequately reflect damage and restoration of the optic nerve. A recent study in patients with NMOSD reported that serum levels of glial fibrillary acidic protein (GFAP) are increased by acute attack, including ON^18^. However, the detailed associations between serum proteins and ON disease characteristics such as severity and prognosis have not been evaluated. Moreover, the significance of serum proteins in patients with ON from etiologies other than NMOSD has yet to be explored.

To this end, we determined if serum proteins were accurate biomarkers for optic nerve damage and restoration in consecutive patients with recent ON. Serum levels of neurofilament light chain (NfL) and GFAP were measured as putative markers for neuronal damage and astrocytic damage, respectively^12,14^. In addition, serum levels of brain-derived neurotrophic factor (BDNF) were measured as a putative marker of restoration due to the potential role of BDNF in regeneration and remyelination^19–22^. We compared these serum proteins by disease group and state (attack vs. remission), and examined their relevance as biomarkers for the severity and prognosis of ON.

## Results

### Patient baseline characteristics

During the study period, we enrolled 507 patients with CNS demyelinating diseases in our prospective registry. Of these patients, we included 60 patients whose last attack was ON in the study (Fig. S1 in the Data Supplement), including 15 patients with AQP4-ON, 14 with MOG-ON, and 31 with DSN-ON. A total of 34 patients were in the attack state (9 AQP4-ON; 9 MOG-ON; 16 DSN-ON). A total of 19 patients (7 AQP4-ON; 6 MOG-ON; 6 DSN-ON) underwent follow-up blood samples 6–12 months after the index attack and were included in the longitudinal analysis. Among the DSN patients, there were three patients with MS who fulfilled the 2017 McDonald criteria^23^, six with chronic recurrent inflammatory optic neuritis^24^, and 22 with single isolated optic neuritis. No patients met the seronegative NMOSD criteria in DSN-ON group^25^.

Baseline characteristics are summarized in Table 1. The proportion of patients with recurrent ON did not significantly differ between disease groups. The number of ON attacks was lower in the DSN-ON group than in the AQP4-ON and MOG-ON groups. The proportion of patients undergoing preventive treatment, including oral steroids, was lower in the DSN-ON group. No significant differences were identified in baseline visual acuity scores between disease groups in either the attack or remission state.

**Table 1:** Patient baseline characteristics at enrollment.

Characteristic	AQP4-ON	MOG-ON	DSN-ON	*p*
	(n = 15)	(n = 14)	(n = 31)	
Age, median (IQR)	45.0 (35.0–61.0)	49.5 (35.5–49.5	38.3 (29.0–56.0)	0.193
Age at onset, median (IQR)	45.0 (30.0–55.6)	40.0 (23.5–58.0)	36.4 (26.6–52.6)	0.410
Female, n (%)	11 (73.3)	8 (57.1)	21 (67.7)	0.642
Recent relapse, n (%)	9 (60.0)	9 (64.3)	16 (51.6)	0.697
Only ON, n (%)	9 (60.0)	11 (78.6)	28 (90.3)	0.054
Recurrent ON, n (%)	10 (66.7)	7 (50.0)	12 (38.7)	0.203
Bilateral ON, n (%)	2 (13.3)	3 (21.4)	4 (12.9)	0.743
No. of ON attacks, median (IQR)	2.0 (1.0–3.0)	2.0 (1.0–3.5)	1.0 (1.0–1.0)	0.018
Disease duration, median (IQR)	4.1 (0.1–6.1)	1.7 (0.3–6.7)	0.6 (0.1–4.8)	0.293
Preventive treatment, n (%)	11 (73.3)	10 (71.4)	3 (9.7)	< 0.001
Steroid, n	8	6	0	
AZA or MMF, n	10	7	1	
Rituximab, n	1	0	0	
Interferon β, n	0	0	2	
Visual acuity at baseline				
Attack state, median (IQR)	0.010 (0.005–0.500)	0.030 (0.005–0.320)	0.100 (0.001–0.500)	0.791
Remission state, median (IQR)	0.200 (0.050–0.630)	0.800 (0.250–1.000)	0.800 (0.250–1.000)	0.241
EDSS at baseline				
Attack state, median (IQR)	4.0 (3.0–4.8)	3.0 (2.5–4.3)	3.0 (1.3–4.0)	0.082
Remission state, median (IQR)	4.0 (1.5–4.7)	1.0 (0.5–3.5)	2.0 (1.0–4.0)	0.053
Days from last attack to blood sampling				
Attack state, median (IQR)	8.0 (3.0–36.0)	11.0 (7.5–24.5)	22.5 (8.0–35.3)	0.474
Remission state, median (IQR)	371.0 (162.0–762.5)	328.0 (211.0–365.0)	269.0 (149.5–1234.5)	0.961

*AQP4-ON* optic neuritis with aquaporin-4 antibody, *AZA* azathioprine, *DSN-ON* double-seronegative optic neuritis, *EDSS* expanded disability status scale, *IQR* interquartile range, *MMF* mycophenolate mofetil, *MOG-ON* optic neuritis with myelin oligodendrocyte glycoprotein antibody, *ON* optic neuritis.

### Serum biomarkers and cross-sectional analysis

The time interval from the last attack to blood sampling was similar across the disease groups (Table 1). Serum damage marker levels, including NfL and GFAP, were significantly positively correlated with age (NfL *r* = 0.424, *p* = 0.001 and GFAP *r* = 0.290, *p* = 0.024) and moderately correlated with one another (*r* = 0.517, *p* < 0.001). Meanwhile, serum levels of the restoration marker BDNF were not significantly correlated with age or damage biomarkers (Table S1 in the Data Supplement). When we compared biomarker levels between patients with first and recurrent ON attacks, there were no significant difference between groups in either the attack or remission state (Table S2 in the Data Supplement).

**Figure 1:**
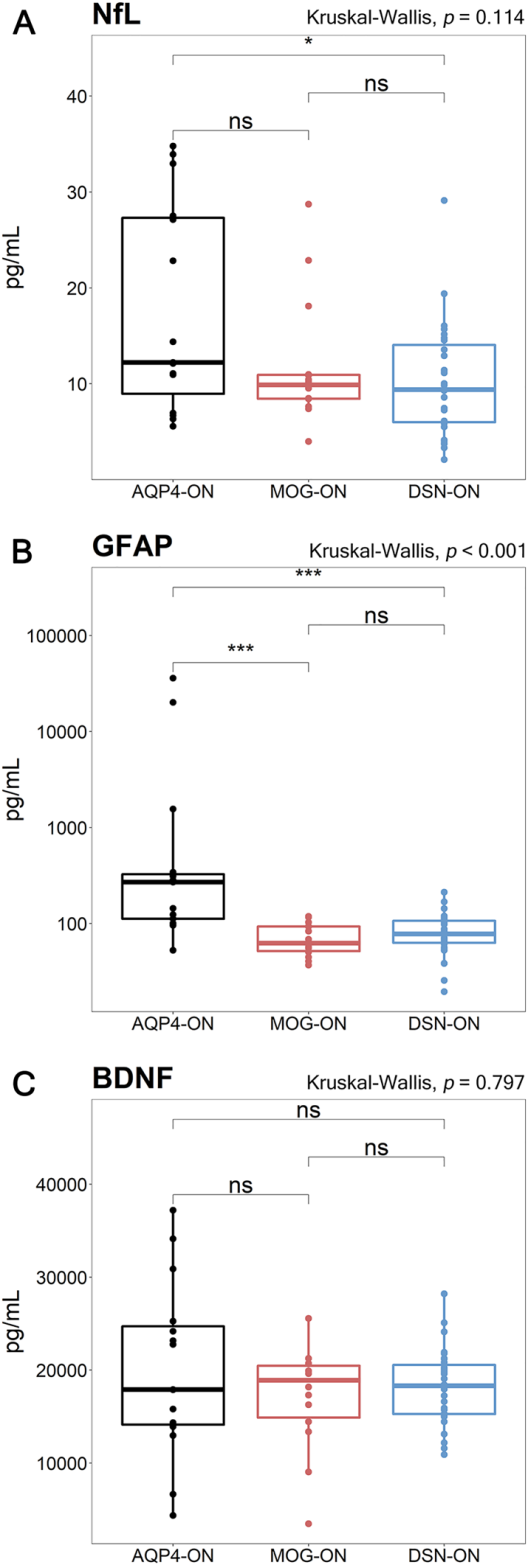
Comparison of serum biomarkers in patients with AQP4-ON, MOG-ON, and DSN-ON. Serum (**A**) NfL, (**B**) GFAP, and (**C**) BDNF levels were compared according to ON etiology at baseline. Boxes represent median and IQR. Posthoc analysis was performed with Bonferroni correction. *p < 0.05, ***p < 0.001. *ns* not significant, *AQP4-ON* optic neuritis with aquaporin-4 antibody, *BDNF* brain-derived natriuretic factor, *DSN-ON* double-seronegative optic neuritis, *GFAP* glial fibrillary acidic protein, *IQR* interquartile range, *MOG-ON* optic neuritis with myelin oligodendrocyte glycoprotein antibody, *NfL* neurofilament light chain, *ON*, optic neuritis.

In disease group analysis (Fig. 1), serum NfL levels were higher in the AQP4-ON group than in the other groups, although this relationship did not reach statistical significance (AQP4-ON, 12.2 [7.0–27.5] vs. MOG-ON, 9.9 [8.2–12.8] vs. DSN-ON, 9.4 [5.9–14.5] pg/mL, *p* = 0.114). Serum GFAP levels in the AQP4-ON group were significantly higher than those of the MOG-ON and DSN-ON groups (AQP4-ON, 269.1 [111.7–342.1] vs. MOG-ON, 62.5 [48.8–95.7] vs. DSN-ON, 77.6 [61.0–109.9] pg/mL, *p* < 0.001). In addition, serum GFAP/NfL levels exhibited a pattern (AQP4-ON, 16.7 [9.1–25.6] vs. MOG-ON, 6.3 [4.5–9.8] vs. DSN-ON, 9.3 [6.0–13.5], *p* = 0.005), similar to serum GFAP levels. Contrastingly, serum BDNF levels did not differ between disease groups. There were no significant differences in biomarker levels between subgroups of the DSN-ON (MS, CRION, and SION; Fig. S2 in the Data Supplement). When subdivided according to disease state (Fig. S3 in the Data Supplement), serum GFAP levels were significantly higher in the AQP4-ON group than in other groups in both the attack and remission states, and the difference was more pronounced in the attack state. In the AQP4 group, serum GFAP levels decreased as the interval between attack and blood sampling increased (Fig. S4 in the Data Supplement).

We subsequently determined if serum biomarkers levels differed between disease states within each disease group in a cross-sectional manner (Fig. S5 in the Data Supplement). We found that serum NfL and GFAP levels were significantly higher in the attack state relative to the remission state in the AQP4-ON group (attack vs. remission, NfL: 27.1 [11.6–33.4] vs. 10.3 [7.3–13.3] pg/mL, *p* = 0.017; GFAP: 281.9 [196.3–10,874.3] vs. 111.7 [98.6–155.4] pg/mL, *p* = 0.021). However, we did not detect significant differences in serum NfL and GFAP levels between disease states in the other groups. Serum BDNF levels were similar between the attack and remission states in all groups.

**Figure 2:**
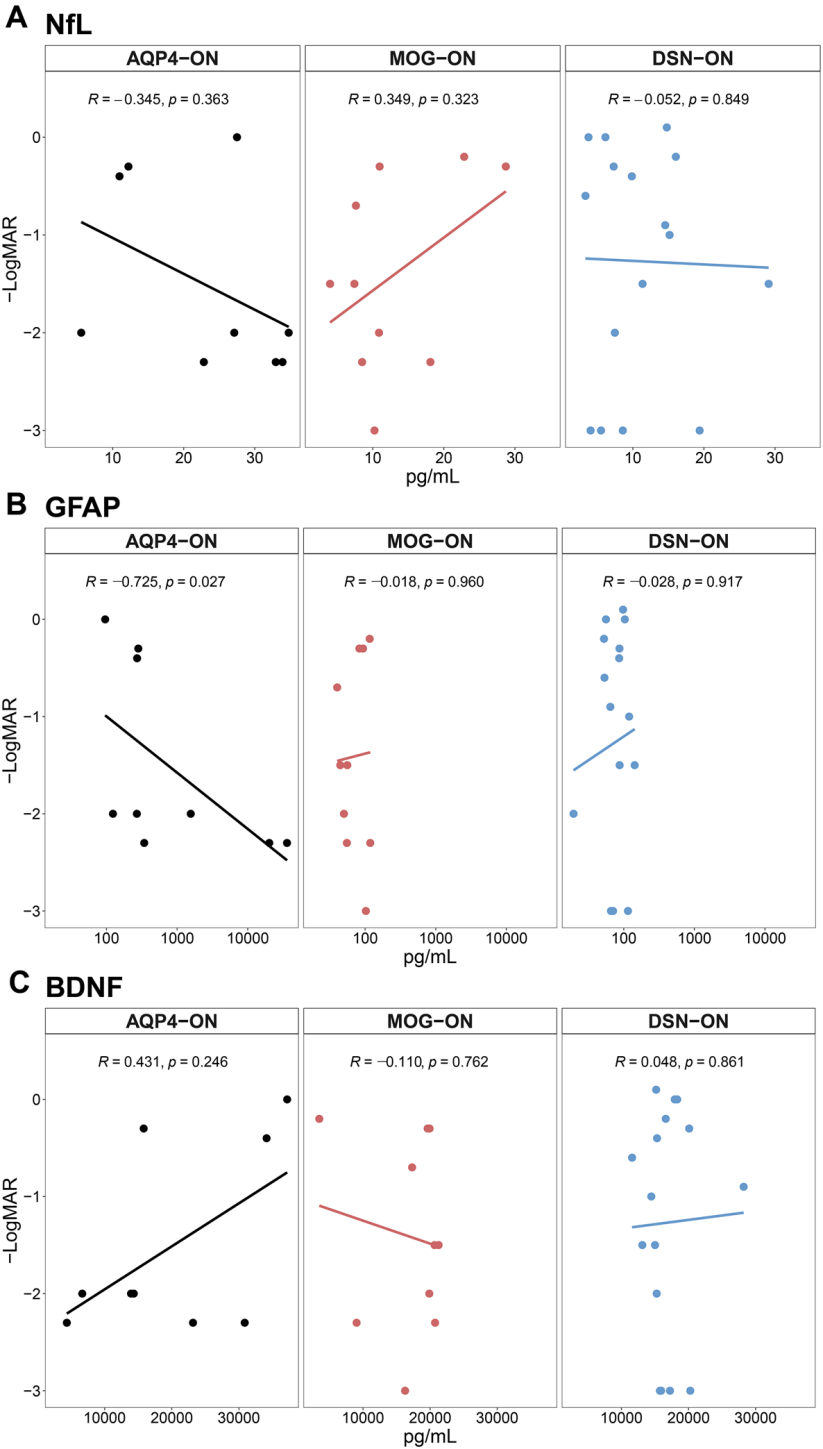
Correlation analyses between visual acuity at attack state and baseline serum biomarkers in patients with AQP4-ON, MOG-ON, and DSN-ON. Correlation between visual acuity (− Logarithm of the Minimum Angle of Resolution) at attack state and serum biomarkers levels at baseline for serum (**A**) NfL, (**B**) GFAP, and (**C**) BDNF in patients with ON. *AQP4-ON* optic neuritis with aquaporin-4 antibody, *BNDF* brain-derived natriuretic factor, *DSN-ON* double-seronegative optic neuritis, *GFAP* glial fibrillary acidic protein, *MOG-ON* optic neuritis with myelin oligodendrocyte glycoprotein antibody, *NfL* neurofilament light chain, *ON* optic neuritis.

For disease severity analysis, serum GFAP levels (*r* = − 0.725, *p* = 0.027), but not serum NfL or BDNF levels, were negatively correlated with visual acuity at attack state in the AQP4-ON group (Fig. 2 for − Logarithm of the Minimum Angle of Resolution [LogMAR]; Fig. S6 in the Data Supplement for untransformed visual acuity). No other significant relationships were observed in the MOG-ON or DSN-ON groups. When we performed severity analysis in all patients, including those in the remission state, we detected no statistically significant correlations between visual acuity and serum biomarkers at baseline (Table S3 in the Data Supplement).

### Longitudinal analysis

**Figure 3:**
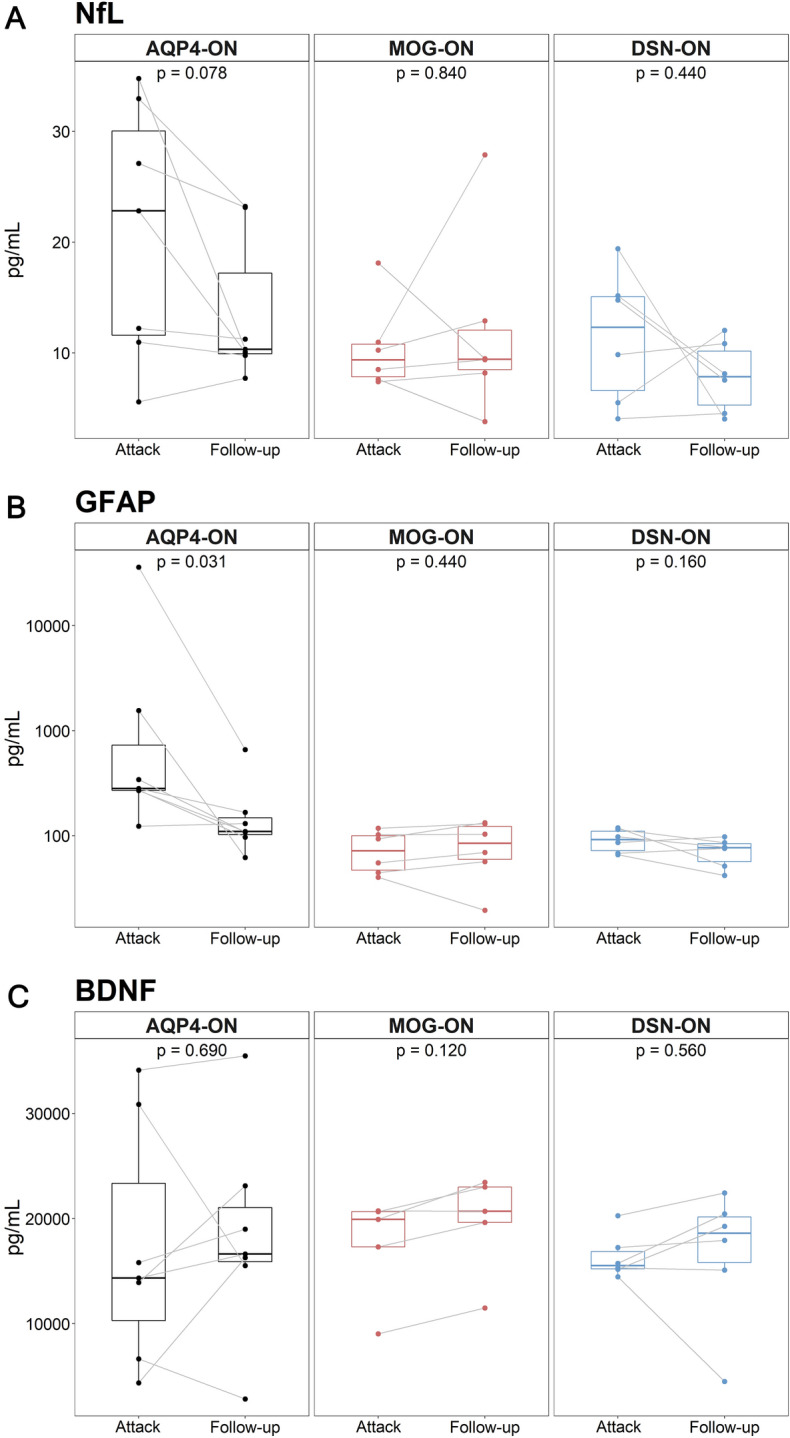
Longitudinal serum biomarker analysis in patients with AQP4-ON, MOG-ON, and DSN-ON. In longitudinal analyses, serum (**A**) NfL, (**B**) GFAP, and (**C**) BDNF levels were compared between baseline (attack state) and follow-up (remission state) in consecutive patients. *AQP4-ON* optic neuritis with aquaporin-4 antibody, *BNDF* brain-derived natriuretic factor, *DSN-ON* double-seronegative optic neuritis, *GFAP* glial fibrillary acidic protein, *MOG-ON* optic neuritis with myelin oligodendrocyte glycoprotein antibody, *NfL* neurofilament light chain.

In the longitudinal analysis (Fig. 3), we compared serum biomarker levels between the attack (baseline) and remission states (6–12 months follow-up) within the same patients. In the AQP4-ON group, serum GFAP levels significantly decreased in the remission state relative to the attack state (281.9 [269.1–1559.0] vs. 109.8 [96.9–167.2] pg/mL; *p* = 0.031). Serum NfL levels also decreased between attack and remission in the AQP4-ON group, but the difference did not reach statistical significance (*p* = 0.078). Serum NfL and GFAP levels in the MOG-ON and DSN-ON groups did not significantly differ between the attack and remission states. Changes in serum BDNF level did not differ between disease states in any of the disease groups.

**Figure 4:**
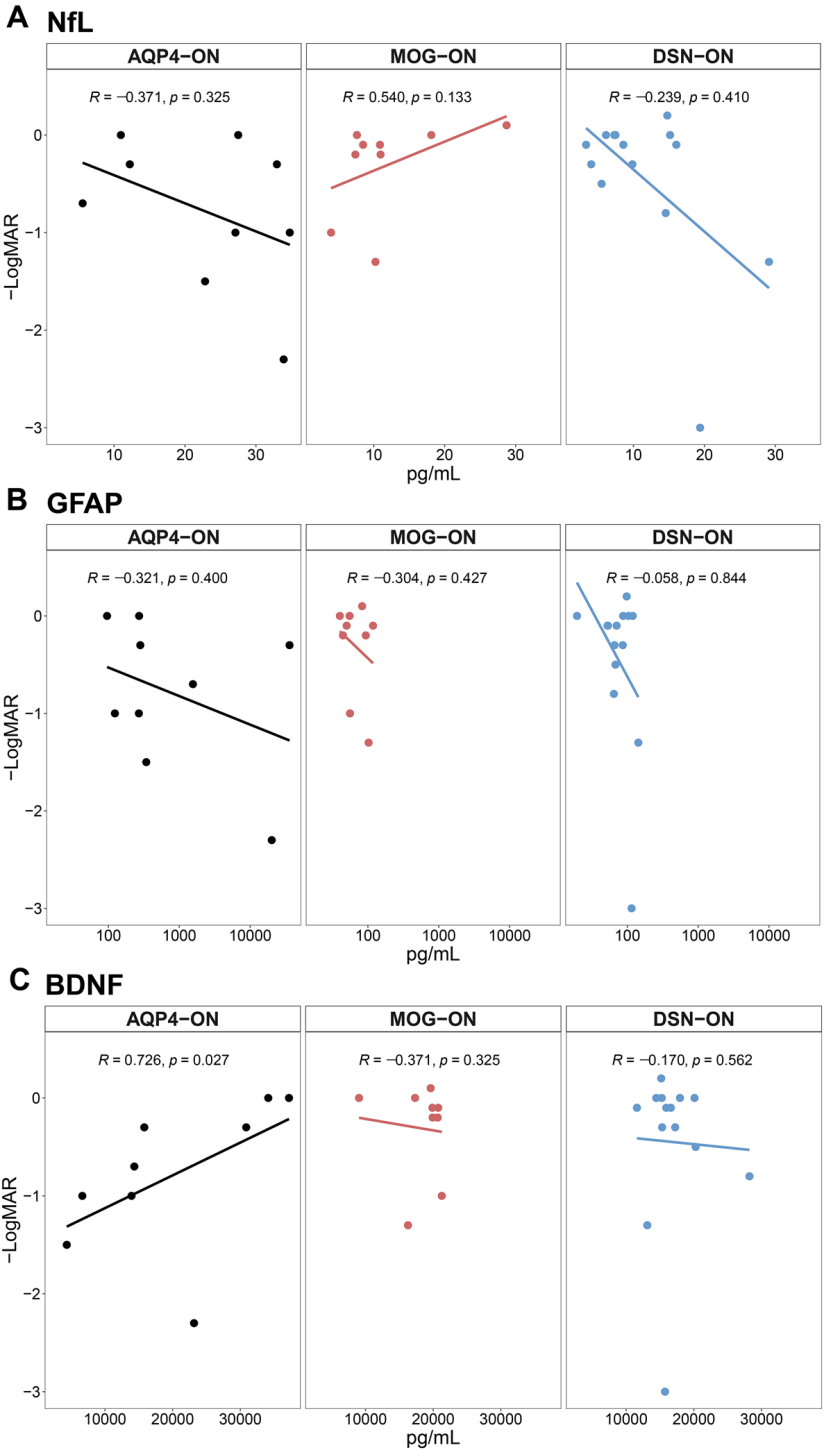
Correlation analyses between visual acuity at remission state and baseline serum biomarkers in patients with AQP4-ON, MOG-ON, and DSN-ON. Correlation between visual acuity (− Logarithm of the Minimum Angle of Resolution) at follow-up (remission state) and serum biomarkers levels at baseline (attack state) for (**A**) NfL, (**B**) GFAP, and (**C**) BDNF in patients with ON. *AQP4-ON* optic neuritis with aquaporin-4 antibody, *BNDF* brain-derived natriuretic factor, *DSN-ON* double-seronegative optic neuritis, *GFAP* glial fibrillary acidic protein, *MOG-ON* optic neuritis with myelin oligodendrocyte glycoprotein antibody, *NfL* neurofilament light chain, *ON* optic neuritis.

To determine the prognostic value of the biomarkers to predict future visual outcomes, we investigated the correlations between the serum biomarkers at baseline (attack state) and follow-up visual acuity (Fig. 4 for − LogMAR; Fig. S7 in the Data Supplement for untransformed visual acuity). Remarkably, serum BDNF was significantly positively correlated with visual acuity at follow-up in the AQP4-ON group (*r* = 0.726, *p* = 0.027). However, this association was not present in the other disease groups. Meanwhile, serum NfL and GFAP levels were not significantly correlated with visual acuity at follow-up in any of the disease groups.

## Discussion

In this prospective study, we demonstrated that serum biomarker levels were reflective of disease status and predictive of prognosis in patients with ON, in which damage occurs in only a small proportion of the CNS. Remarkably, serum GFAP levels were significantly correlated with severe visual impairment during the attack state in the AQP4-ON group. On the other hand, serum BDNF levels were positively associated with improved future visual outcome in the AQP4-ON group.

Serum GFAP levels were significantly higher in patients with AQP4-ON than in the other disease groups. In addition, serum GFAP levels were significantly elevated during ON attacks, and were significantly negatively correlated with visual function during the acute attack state in the AQP4-ON group. GFAP is a cytoskeletal scaffolding protein expressed in astrocytes^26^. AQP4 is located in the endfeet of astrocytes^27^. Serum GFAP levels increase during the acute attack and are reflective of disease severity in patients with seropositive NMOSD with anti-AQP4 antibodies (AQP4-Ab)^14,28–31^. Our findings are consistent with these results, and further suggest that GFAP leakage upon astrocyte damage, even that induced by ON attacks, is sufficiently reflected in the serum. Increased serum GFAP levels during AQP4-ON attacks have been reported in studies using conventional ELISA techniques in cross-sectional analyses^32,33^. However, no prior studies have identified correlations between visual function and serum GFAP levels, or performed analyses in a longitudinal cohort. The present study thus presents novel findings, while also further supporting the utility of serum GFAP as a disease biomarker in patients with AQP4-ON.

Serum NfL levels did not significantly differ across disease groups. Moreover, they showed only a modest difference according to disease state (attacks vs. remission) within the AQP-4 ON group, while no significant differences in the other ON groups. Serum NfL levels also did not correlate with the degree of visual symptoms. Based on these findings, serum NfL may not be a useful biomarker in patients with ON. NfL is an axonal cytoskeletal protein that is released upon neuroaxonal damage regardless of causes^12^. Therefore, it is reasonable serum NfL was not specific enough to distinguish a certain type of ON from the others. The lack of strong association between serum NfL levels and disease activity (attacks) or severity (visual function) is noteworthy considering that NfL is a promising biomarker reflecting disease status in various neurological diseases^12,14,30,31,34^. The size of the optic nerve could be too small to manifest correlations between serum NfL and inflammatory ON.

In the ON groups other than AQP4-ON, both GFAP and NfL were not reflective of disease stage or decreased visual acuity during the acute attack phase, and so were not identified as a useful biomarker. Because neither patient with MOG-ON nor DSN-ON have direct pathogenic antibodies against astrocytes, GFAP is less likely to be relevant to these pathologies. In addition, less-severe ON in the MOG- and DSN-ON groups relative to that of the AQP4-ON group may have resulted in negative correlations with serum GFAP and NfL levels^35^.

Recent studies have investigated the role of serum NfL and GFAP levels as a biomarker in patients with AQP4-Ab+ NMOSD and MOGAD^14,18,29–31,36–39^. Both biomarkers have been reported to increase upon relapses and decrease over time in both diseases^18,30,31,39^. However, the degree of increase may be different between biomarkers according to pathogenic mechanisms. In patients with AQP4-Ab+ NMOSD, serum GFAP levels increases more prominently than serum NfL levels during relapses. Several studies have shown that the serum GFAP/NfL ratio is more closely associated with relapses in patients with AQP4-Ab+ NMOSD compared to either serum levels of GFAP or NfL alone^14,30,38^. Accordingly, serum GFAP/NfL ratios were higher in patients with AQP-ON than others in this study. On the other hand, in patients with MOGAD, the significance of serum NfL or GFAP levels is less established. One study reported that serum levels of NfL and GFAP increased during relapse, with NfL increasing more remarkably during severe attacks in patients with MOGAD^29^. However, it has also been shown that neither serum GFAP nor NfL levels increases during relapses, while only serum tau level does^31^. Another study showed that NfL increases during relapses without an increase in GFAP^40^. These inconsistent results in MOGAD may have arisen from the absence of direct target antibodies against astrocytes and less attack severity than in AQP4-Ab+ NMOSD. This study is noteworthy in that it is the first study to describe similar trends as above in patients with ON. In AQP4-ON, serum GFAP levels increased even with damage in a small proportion of the CNS, suggesting their role as a biomarker. In MOG-ON, however, both serum NfL and GFAP levels failed to clearly demonstrate their usefulness as biomarkers.

Serum BDNF levels during acute ON attacks were positively correlated with improved visual function during the recovery phase in patients with AQP4-ON. BDNF is a member of the neurotrophin family in the brain, and plays important roles in neuroprotection and remyelination in neurological diseases, especially in inflammatory conditions^19,20^. BDNF has also been reported to have a protective role in damage to the optic nerve in a mouse model^41^. However, no prior studies have evaluated the implication of BDNF in patients with inflammatory ON. Our findings suggest that serum BDNF is a putative prognostic biomarker in patients with AQP4-ON.

The predictive value of BDNF was not significant in the other ON groups. The source of BDNF in the CNS, which could potentially be neurons, astrocytes, or immune cells, could differ between pathologies^42,43^. In contexts in which endogenous BDNF should be increased, such as CNS damage states, astrocytes could serve as the primary source of BDNF^19,43^. In a mouse model of experimental autoimmune encephalomyelitis, depletion of astrocyte-specific BDNF increases disease severity^19^. In addition, astrocyte-derived BDNF promotes oligodendrogenesis in ischemic white matter damage and demyelinating models^20–22^. Therefore, the abundance and soundness of astrocytes may be more important than that of other BDNF sources for recovery from inflammatory attacks. Direct astrocytic damage in patients with anti-AQP4 antibodies could have accentuated the role of BDNF in recovery from inflammation in AQP4-ON patients in the present study.

Although BDNF was indicated as a prognostic biomarker, it was not significantly correlated with damage biomarkers. Because serum NfL and GFAP levels reflect not only reflect disease severity, which could trigger BDNF release^44,45^, but also reflect the degree of BDNF source depletion, straightforward correlations might not have been present. Contrastingly, negative correlations between age and serum BDNF levels, which have been suggested in studies of age-dependent cognitive decline and synaptic loss^46^, were also not detected. Varying disease states in patients, such as attack and remission, could have attenuated the relationship between age and serum BDNF levels.

This study did not include a healthy control to compare serum biomarkers of the disease group. Although direct comparisons are difficult due to the possibility of inter-rater variability, it can be compared to the values of several previously published studies (Table S4 in the Data supplement). The median serum NfL and GFAP level of healthy controls in the same institution as this study was 10.2 [8.0–14.0] and 98.9 [61.3–142.4], respectively^30^. In other published studies^18,47,48^, the median serum NfL and GFAP levels of healthy controls ranged from 6.3–22.9 and 60.2–71.3, respectively. Compared to these results, serum NfL was not elevated in any of the three ON groups; serum GFAP was elevated in AQP4-ON but not in MOG-ON or DSN-ON.

The study has several limitations that should be acknowledged to prevent its over interpretation. First, we included patients with ON that previously experienced attacks in the brain or spinal cord. Therefore, clinical attacks before the index ON event could have affected our results. However, the median time interval from other attacks and index ON was > 1 year (median [IQR]: 1709.0 [1221.0–2382.0] days for AQP4-ON; 628.0 [435.5–2954.5] days for MOG-ON; 1189.0 [797.5–1478.0] days for DSN-ON), suggesting that the impact of previous attacks on serum biomarker levels would not be significant. Second, we analyzed serum biomarkers in combined patients with first and recurrent ON attack. Thus, we cannot negate the cumulative effect of multiple ON attacks on serum biomarker levels. However, it should be noted that there was no significant difference in biomarker levels between the single attack and recurrent attack groups (Table S2 in the Data Supplement). In addition, since baseline visual acuity before ON attack was unknown, the relationship between attack severity (degree of worsening of visual acuity) and biomarkers could not be evaluated. Third, we did not consider visual outcome variables other than visual acuity, for example, optical coherence tomography (OCT), to evaluate ON severity. OCT is a sensitive tool to evaluate the degree of axonal loss in ON associated with CNS demyelinating diseases^49,50^. Further studies are needed to investigate the clinical relevance of serum biomarkers that correlate with OCT changes. Fourth, we used a test based on fixed cell-based assay to diagnose anti-AQP4 and anti-MOG antibody. Without a confirmatory live cell-based assay, it is possible that the diagnostic test may have misclassified several patients with MOG-ON as the DSN-ON group. Finally, this was a single-center study conducted in a single ethnicity, and the sample size was small. All of these caveats could potentially compromise the generalizability of our findings. Therefore, the results should be interpreted cautiously.

## Conclusion

In this prospective longitudinal cohort study, serum GFAP levels reflected disease status and severity in AQP4-ON, while serum BDNF showed potential as a prognostic marker for patients with AQP4-ON. These findings suggest that serum CNS proteins could serve as biomarkers in patients with ON, warranting larger future studies to further evaluate their utility.

## Methods

### Patients

Beginning in June 2018, we prospectively recruited adult patients with CNS demyelinating disease that visited the Department of Neurology at the Asan Medical Center (Seoul, Korea). We simultaneously enrolled consecutive patients and secured informed consent regardless of the presence of clinical events. All patients underwent tests for antibodies against AQP4 and MOG using a cell-based assay (Euroimmun, Lübeck, Germany)^51,52^.

In the present study, we considered patients that were enrolled between June 2018 and April 2020, analyzing the data from patients whose most recent attack was ON. We included patients with previous history of myelitis or brain lesions prior to the index ON attack. However, we excluded patients with a simultaneous attack in other CNS sites (brain or spinal cord), even if the most recent attack involved ON. An expert ophthalmologist diagnosed ON by ophthalmologic examination and orbital magnetic resonance imaging. We grouped the patients with ON by antibody status: ON with anti-AQP4 antibody (AQP4-ON), ON with anti-MOG antibody (MOG-ON), and double-seronegative ON (DSN-ON).

### Measurements

We defined the attack state as occurring within 2 months from the last ON attack^14,53^, and the remission state as a partial or complete recovery period at least 2 months after the last attack. Following the study protocol, we performed initial blood sampling when the patients were enrolled in the study regardless of disease state. For patients enrolled in the attack state, if they consented, we collected follow-up blood samples after 6–12 months. We examined the patients’ visual acuity (Snellen chart) and Expanded Disability Status Scale (EDSS) score at enrollment and follow-up. Visual acuity at remission was defined as the value at the follow-up sampling 6–12 months after an attack. For measurement of visual acuity, we evaluated the affected eye in cases of unilateral ON attack, and the more severe eye in cases of bilateral ON attack.

### Biomarker analysis

Patient blood samples were collected and stored at − 80 °C following standardized procedures^54^. Samples were thawed immediately before analysis. Serum concentrations of NfL, GFAP, and BDNF were measured by an investigator blinded to the patients’ clinical information in duplicate using a Simoa HD-1 Analyzer (Quanterix, Billerica, MA, USA).

The limits of quantification were 0.241 pg/mL for NfL, 0.467 pg/mL for GFAP, and 0.0293 pg/mL for BDNF when compensated with a four-fold sample dilution for NfL and GFAP, and a 500-fold sample dilution for BDNF. All results were above the detection limit. The mean intra-assay coefficients of variation for NfL, GFAP, and BDNF levels were 3.9%, 3.2%, and 3.2%, respectively. All intra-assay duplicate coefficients of variation for the samples were < 20%.

### Statistical analysis

First, we evaluated correlations between serum protein levels and age in all patients at baseline and between each biomarker level in a cross-sectional manner. In addition, we compared baseline serum biomarker levels between patients with first ON attack and those with recurrent ON attacks by pooling disease groups to determine if serum levels of biomarkers differed between the single- and multiple-attack groups. Subsequently, we compared serum protein levels among disease groups (AQP4-ON, MOG-ON, DSN-ON), and protein levels between disease states (attack vs. remission) within each disease group. We also evaluated the correlations between serum protein levels and visual acuity for disease severity analysis. In addition, we compared GFAP/NfL ratio among ON group, which appeared to be a more specific marker of astrocytopathy than GFAP alone.

Subsequently, we performed a longitudinal analysis in patients who were enrolled during the attack state and underwent a follow-up evaluation in the remission state after 6–12 months. We compared serum biomarker levels between the attack state and follow-up evaluation in consecutive patients. Finally, we evaluated the correlations between serum protein levels and future visual outcome (visual acuity). For this analysis, visual acuity was converted to LogMAR to perform the calculation.

We performed the chi-square or Kruskal–Wallis test (Mann–Whitney U test by Bonferroni correction for posthoc analysis) to compare the variables among the disease groups. We conducted a Wilcoxon signed-rank test to compare serum biomarkers at baseline and follow-up in consecutive patients. We calculated Spearman’s correlation coefficients to evaluate the relationships between serum biomarkers and the relationships between serum biomarkers and clinical variables (visual acuity and EDSS scores). Variables with two-tailed *p* < 0.05 were considered significant. We performed all statistical analyses with either SPSS version 22.0 (SPSS Inc., Chicago, IL, USA) or R version 4.0.0 (R Foundation for Statistical Computing, Vienna, Austria).

### Ethical statement

Institutional Review Board of Asan Medical Center approved the study (No. 2018-0653), and we obtained written informed consent from all study participants. All methods were carried out in accordance with relevant guidelines and regulations.

## Supplementary Information

Supplementary Legends.

Supplementary Figure S1.

Supplementary Figure S2.

Supplementary Figure S3.

Supplementary Figure S4.

Supplementary Figure S5.

Supplementary Figure S6.

Supplementary Figure S7.

Supplementary Tables.

## Data Availability

Processed data used in this study are available from the corresponding author, Eun-Jae Lee, on request from qualified investigators.
