# Supplementary material for: Serum proteins for monitoring and predicting visual function in patients with recent optic neuritis
Source: Sci Rep. 2023 Apr 5;13:5609. doi: 10.1038/s41598-023-32748-5 (PMC10076295; doi:10.1038/s41598-023-32748-5)
Supplement: Supplementary file 1 — Supplementary Legends. [file 41598_2023_32748_MOESM1_ESM.docx]

**Figure S1.** Flow diagram of patient inclusion

Baseline refers to the time when the patients enrolled in the study, and follow-up refers to patients that were in an attack state at baseline and underwent blood sampling after 6−12 months. AQP4-ON, optic neuritis with aquaporin-4 antibody; CNS, central nervous system; DSN-ON, double-seronegative optic neuritis; MOG-ON, optic neuritis with myelin oligodendrocyte glycoprotein antibody.

**Figure S2.** Comparison of serum biomarkers across the subgroup of patients with DSN-ON

Serum (A) NfL, (B) GFAP, and (C) BDNF levels were compared based on subgroup of DSN-ON. Boxes represent median and IQR. BDNF, brain-derived natriuretic factor; CRION, chronic relapsing inflammatory optic neuropathy; DSN-ON, double-seronegative optic neuritis; GFAP, glial fibrillary acidic protein; IQR, interquartile range; SION, single isolated optic neuritis (SION); NfL, neurofilament light chain; MS, multiple sclerosis; ON, optic neuritis.

**Figure S3.** Comparison of serum biomarkers in patients with AQP4-ON, MOG-ON, and DSN-ON at attack and remission states

Serum (A) NfL, (B) GFAP, and (C) BDNF levels were compared according to ON etiology at attack and remission states. Boxes represent median and IQR. Posthoc analysis was performed with Bonferroni correction. *p < 0.05, ***p < 0.001, ns, not significant. AQP4-ON, optic neuritis with aquaporin-4 antibody; BDNF, brain-derived natriuretic factor; DSN-ON, double-seronegative optic neuritis; GFAP, glial fibrillary acidic protein; IQR, interquartile range; MOG-ON, optic neuritis with myelin oligodendrocyte glycoprotein antibody; NfL, neurofilament light chain; ON, optic neuritis.

**Figure S4.** Correlation analyses between the interval between the attack and blood sampling date and serum biomarkers levels

Correlation between the interval between the attack and blood sampling date and biomarkers levels for (A) NfL, (B) GFAP, and (C) BDNF in patients with AQP4-ON, MOG-ON, and DSN-ON. AQP4-ON, optic neuritis with aquaporin-4 antibody; BNDF, brain-derived natriuretic factor; DSN-ON, double-seronegative optic neuritis; GFAP, glial fibrillary acidic protein; MOG-ON, optic neuritis with myelin oligodendrocyte glycoprotein antibody; NfL, neurofilament light chain; ON, optic neuritis.

**Figure S5.** Cross-sectional serum biomarker analysis in patients with AQP4-ON, MOG-ON, and DSN-ON

Serum (A) NfL, (B) GFAP, and (C) BDNF levels were compared between attack and remission states in all patients. AQP4-ON, optic neuritis with aquaporin-4 antibody; BNDF, brain-derived natriuretic factor; DSN-ON, double-seronegative optic neuritis; GFAP, glial fibrillary acidic protein; MOG-ON, optic neuritis with myelin oligodendrocyte glycoprotein antibody; NfL, neurofilament light chain.

**Figure S6.** Correlation analyses between visual acuity at attack state and baseline serum biomarkers in patients with AQP4-ON, MOG-ON, and DSN-ON

Correlation between visual acuity at attack state and serum biomarkers levels at baseline for serum (A) NfL, (B) GFAP, and (C) BDNF in patients with ON. AQP4-ON, optic neuritis with aquaporin-4 antibody; BNDF, brain-derived natriuretic factor; DSN-ON, double-seronegative optic neuritis; GFAP, glial fibrillary acidic protein; MOG-ON, optic neuritis with myelin oligodendrocyte glycoprotein antibody; NfL, neurofilament light chain; ON, optic neuritis.

**Figure S7.** Correlation analyses between visual acuity at remission state and baseline serum biomarkers in patients with AQP4-ON, MOG-ON, and DSN-ON

Correlation between visual acuity at follow-up (remission state) and serum biomarkers levels at baseline (attack state) for (A) NfL, (B) GFAP, and (C) BDNF in patients with ON. AQP4-ON, optic neuritis with aquaporin-4 antibody; BNDF, brain-derived natriuretic factor; DSN-ON, double-seronegative optic neuritis; GFAP, glial fibrillary acidic protein; MOG-ON, optic neuritis with myelin oligodendrocyte glycoprotein antibody; NfL, neurofilament light chain; ON, optic neuritis.
