# Supplementary figures and images for: Serum proteins for monitoring and predicting visual function in patients with recent optic neuritis
Source: Sci Rep. 2023 Apr 5;13:5609. doi: 10.1038/s41598-023-32748-5 (PMC10076295; doi:10.1038/s41598-023-32748-5)

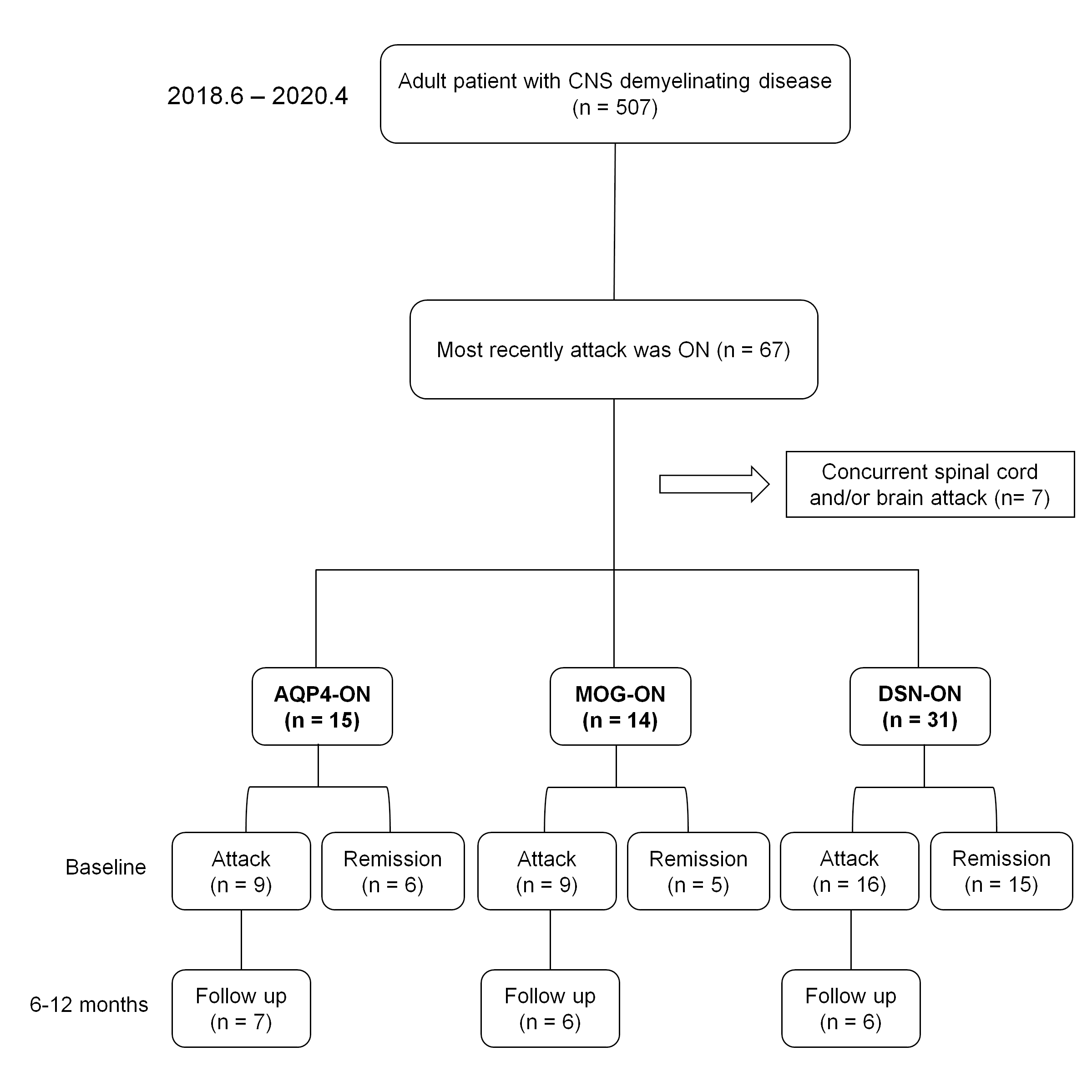

Supplement: Supplementary file 2 — Supplementary Figure S1. [file 41598_2023_32748_MOESM2_ESM.tif]

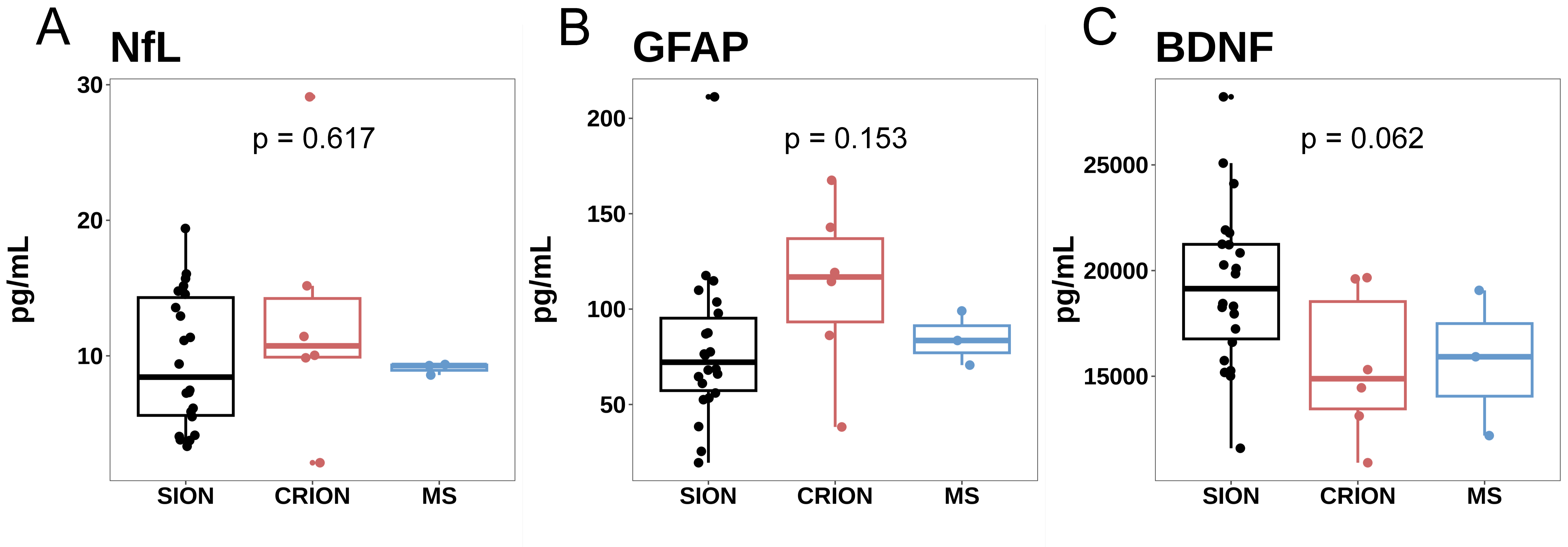

Supplement: Supplementary file 3 — Supplementary Figure S2. [file 41598_2023_32748_MOESM3_ESM.tif]

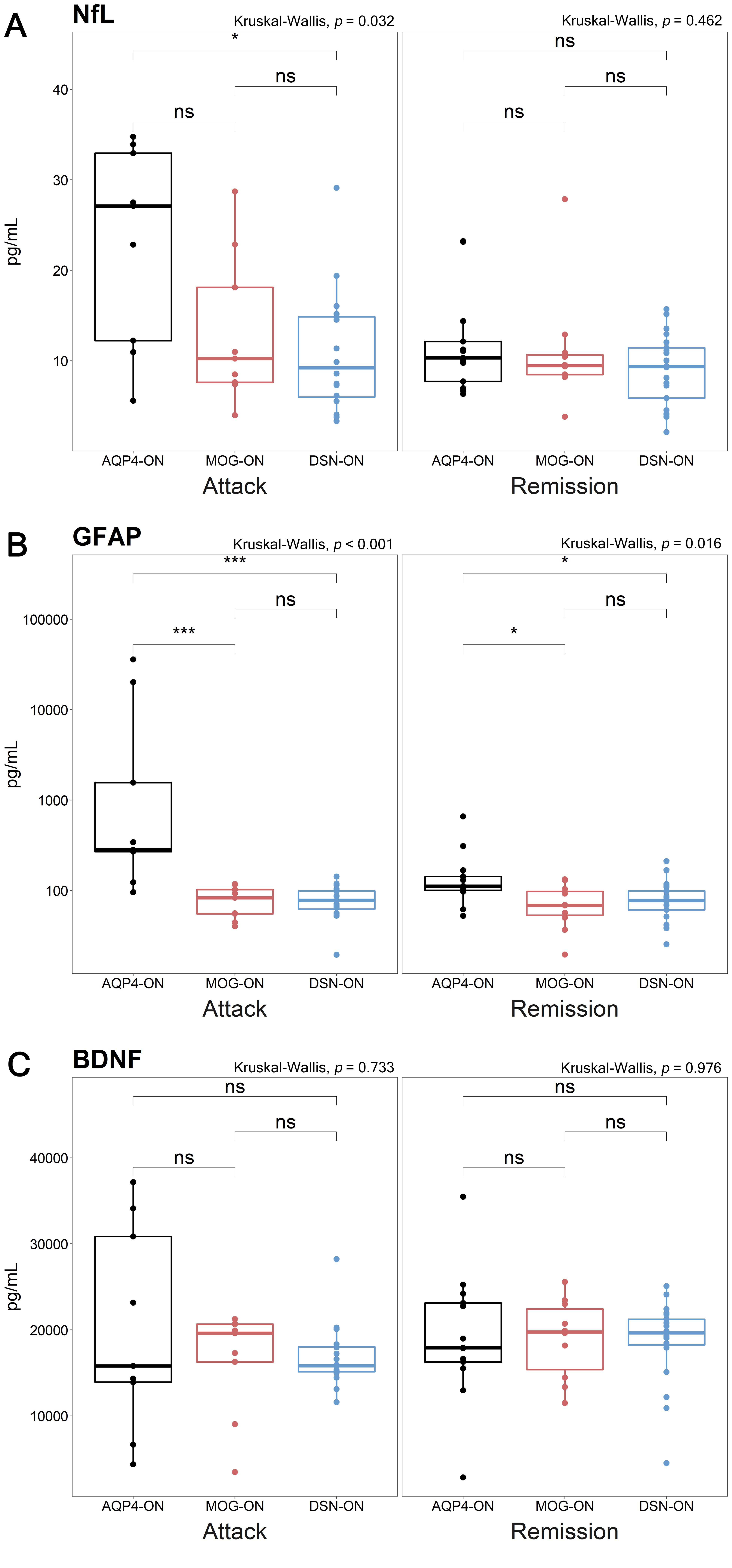

Supplement: Supplementary file 4 — Supplementary Figure S3. [file 41598_2023_32748_MOESM4_ESM.tif]

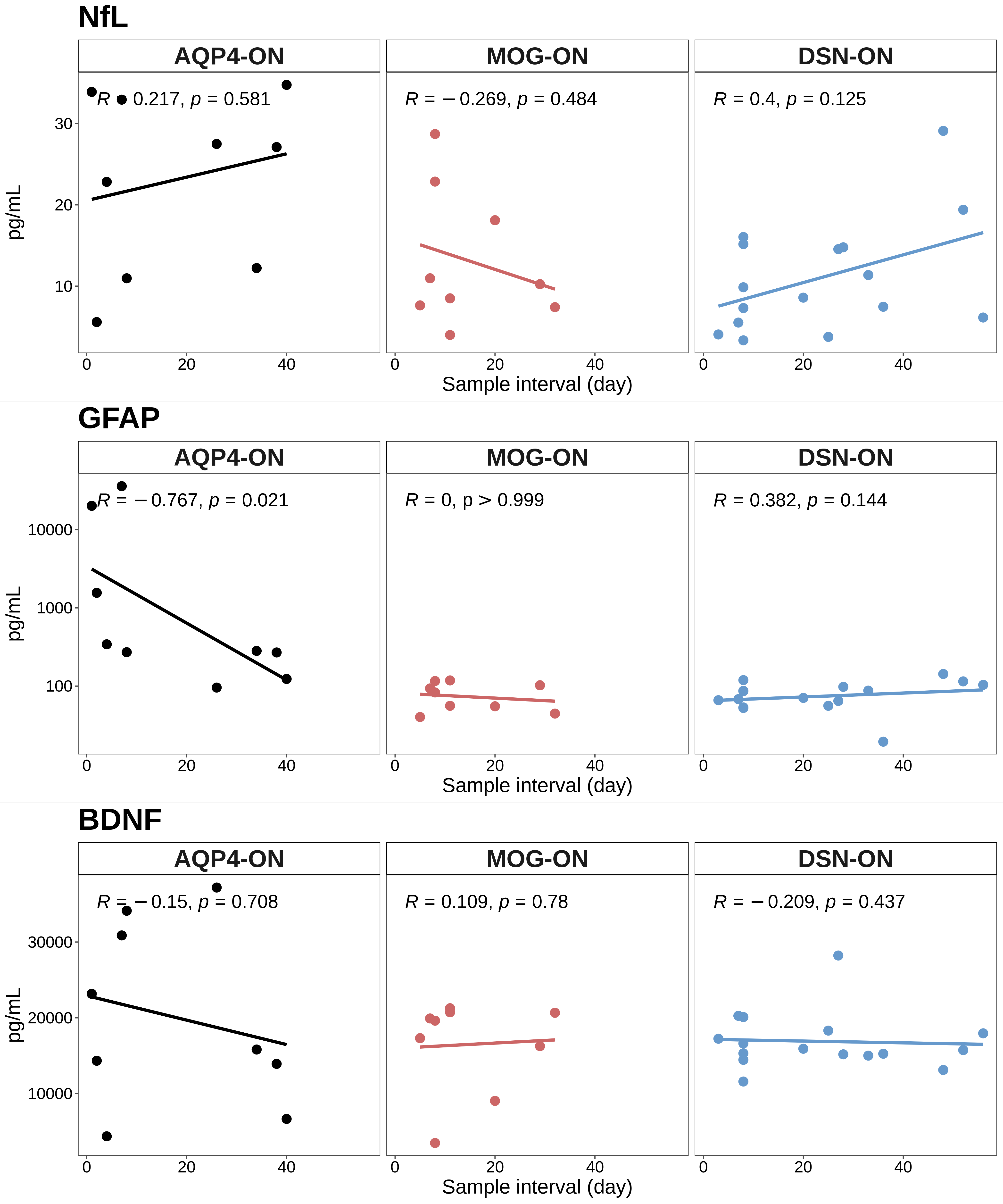

Supplement: Supplementary file 5 — Supplementary Figure S4. [file 41598_2023_32748_MOESM5_ESM.tif]

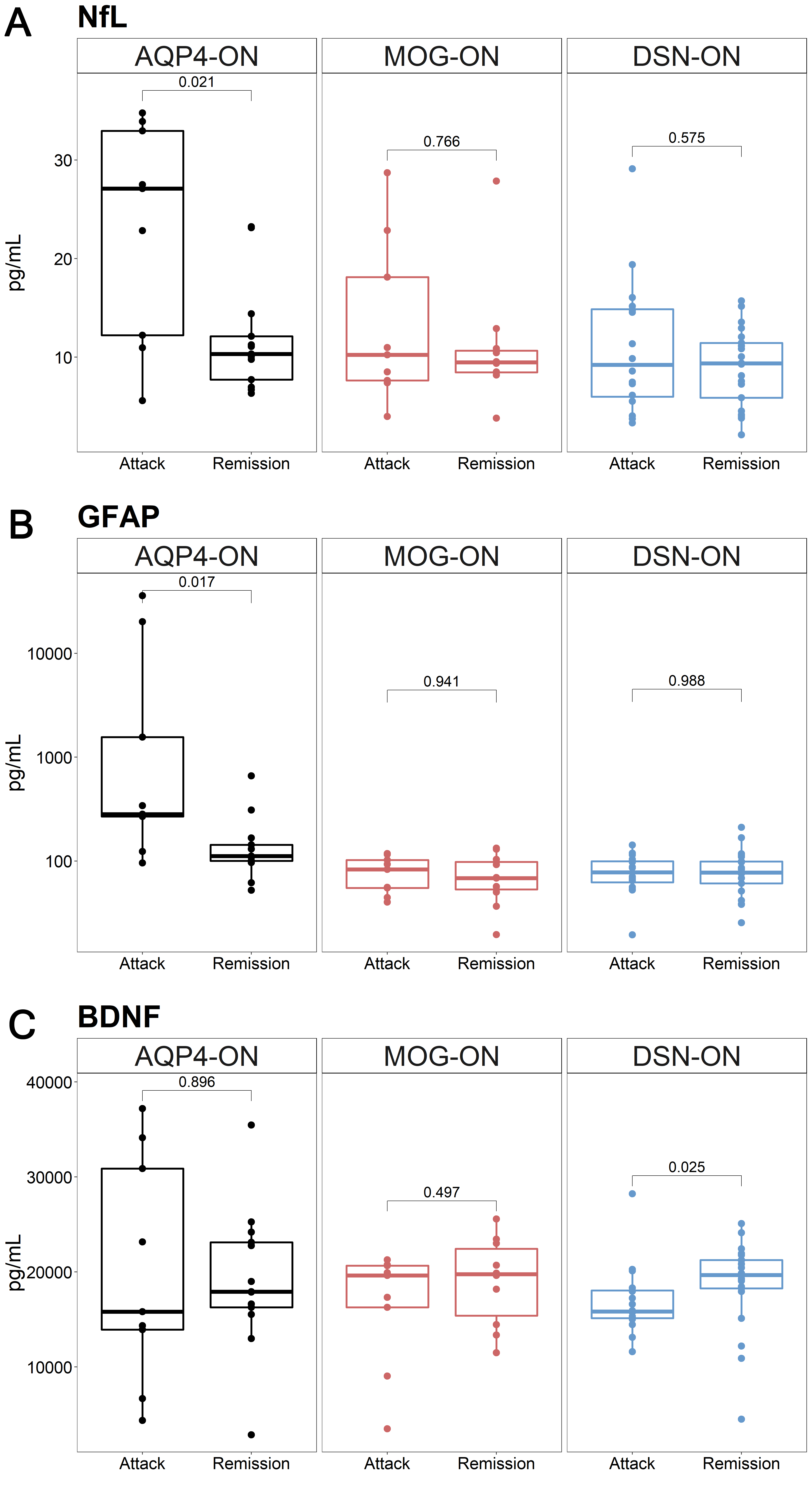

Supplement: Supplementary file 6 — Supplementary Figure S5. [file 41598_2023_32748_MOESM6_ESM.tif]

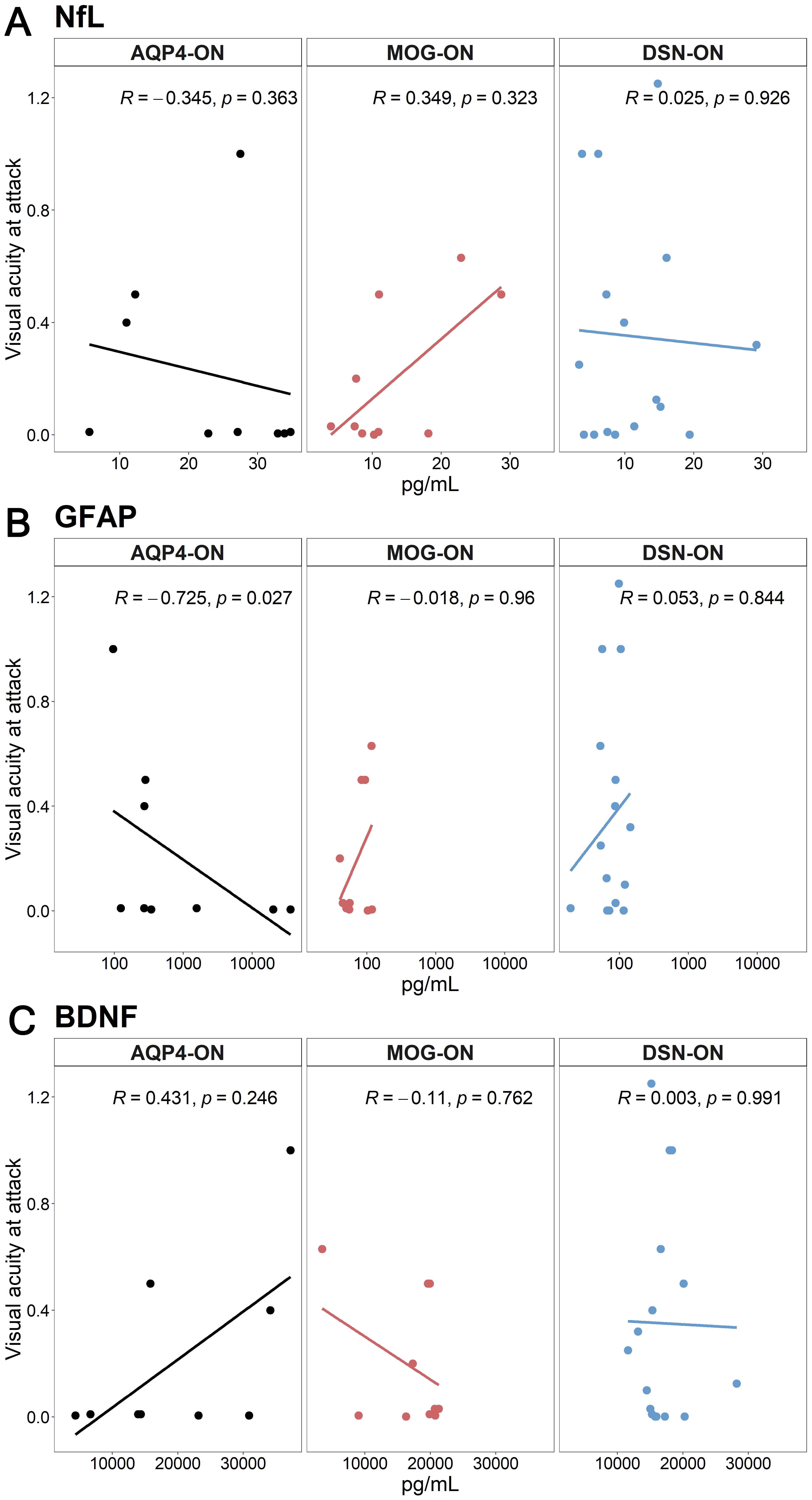

Supplement: Supplementary file 7 — Supplementary Figure S6. [file 41598_2023_32748_MOESM7_ESM.tif]

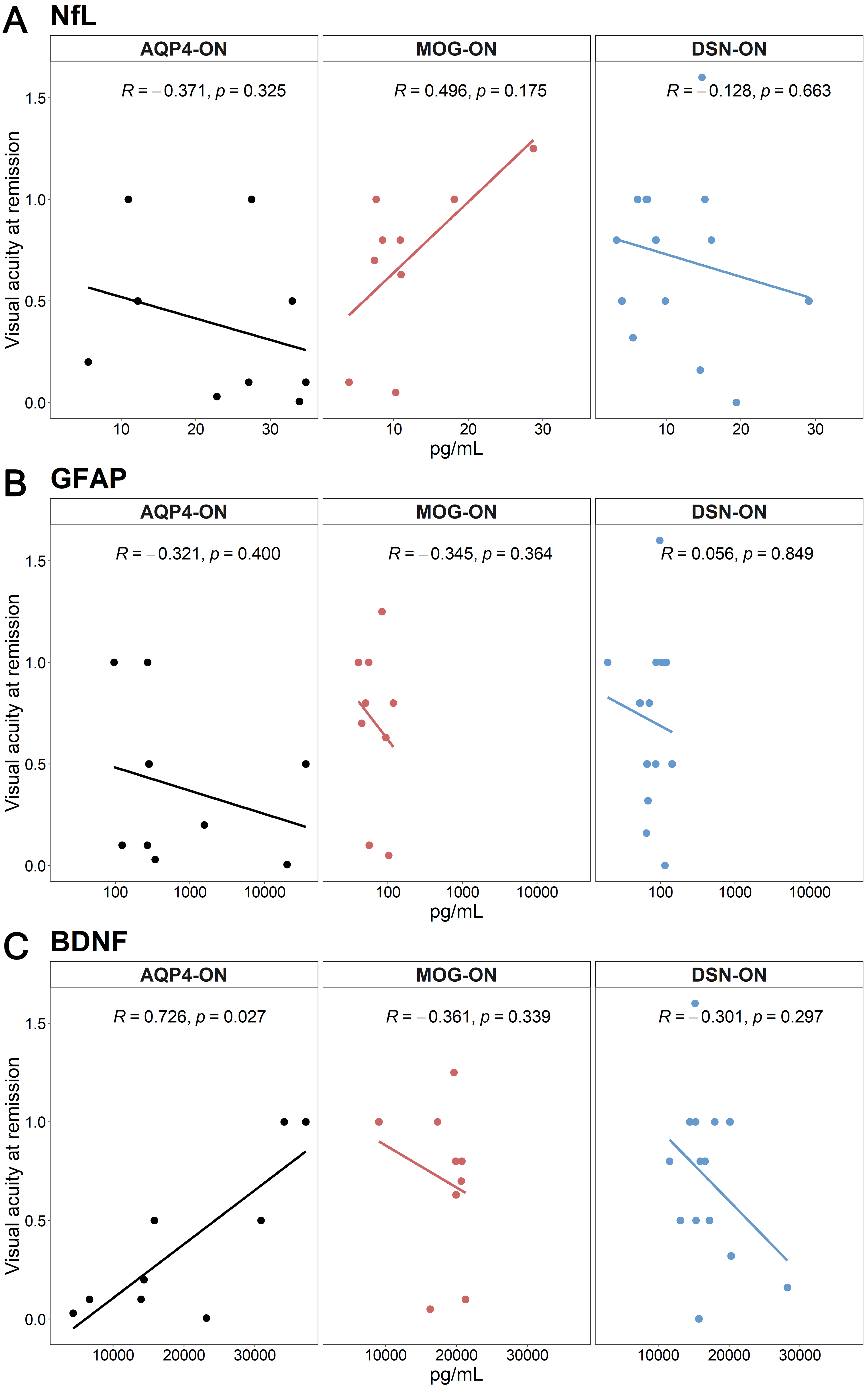

Supplement: Supplementary file 8 — Supplementary Figure S7. [file 41598_2023_32748_MOESM8_ESM.tif]
