# Supplementary material for: Serum proteins for monitoring and predicting visual function in patients with recent optic neuritis
Source: Sci Rep. 2023 Apr 5;13:5609. doi: 10.1038/s41598-023-32748-5 (PMC10076295; doi:10.1038/s41598-023-32748-5)
Supplement: Supplementary file 9 — Supplementary Tables. [file 41598_2023_32748_MOESM9_ESM.docx]

**Table S1.** Correlation of serum biomarkers with visual acuity and other biomarkers at patient enrollment

|  | **Age** | **NfL** | **GFAP** |
| --- | --- | --- | --- |
| NfL | 0.424^**^ |  |  |
| GFAP | 0.290^*^ | 0.517^***^ |  |
| BDNF | 0.042 | −0.056 | −0.113 |

BDNF, brain-derived neurotrophic factor; GFAP, glial fibrillary acidic protein; NfL, neurofilament light chain. *p < 0.05, **p < 0.01, ***p < 0.001

**Table S2.** Comparison of serum biomarkers between first and recurrent attacks at baseline

|  | **First attack** | **Recurrent attack** |  |
| --- | --- | --- | --- |
| **Attack state** | n = 24 | n = 10 | p |
| NfL, median (IQR) | 13.0 (7.3–22.0) | 10.4 (7.0–24.4) | 0.838 |
| GFAP, median (IQR) | 87.3 (58.2–115.8) | 130.5 (78.6–646.3) | 0.101 |
| BDNF, median (IQR) | 16438.4 (14593.5–19980.5) | 17866.7 (14035.4–20868.0) | 0.752 |
| **Remission state** | n = 7 | n = 19 |  |
| NfL, median (IQR) | 11.7 (8.0–14.1) | 9.4 (6.7–11.1) | 0.364 |
| GFAP, median (IQR) | 73.0 (66.6–127.1) | 92.5 (52.4–114.5) | 0.534 |
| BDNF, median (IQR) | 21583.4 (18908.3–25206.0) | 19664.0 (17881.0–21781.6) | 0.279 |

BDNF, brain-derived neurotrophic factor; GFAP, glial fibrillary acidic protein; IQR, interquartile range; NfL, neurofilament light chain.

**Table S3.** Correlation between visual acuity and serum biomarkers at patient enrollment in each disease group

|  | **AQP4-ON** | |  | **MOG-ON** | |  | **DSN-ON** | |
| --- | --- | --- | --- | --- | --- | --- | --- | --- |
|  | *r* | *P* |  | *r* | *p* |  | *r* | *p* |
| NfL | -0.270 | 0.330 |  | 0.119 | 0.699 |  | -0.016 | 0.933 |
| GFAP | -0.455 | 0.089 |  | -0.047 | 0.879 |  | 0.023 | 0.900 |
| BDNF | 0.233 | 0.403 |  | 0.127 | 0.679 |  | 0.077 | 0.681 |

AQP4-ON, optic neuritis with aquaporin-4 antibody; BDNF, brain-derived neurotrophic factor; DSN-ON, double-seronegative optic neuritis; GFAP, glial fibrillary acidic protein; MOG-ON, optic neuritis with myelin oligodendrocyte glycoprotein antibody; NfL, neurofilament light chain.

**Table S4.** Comparison of serum biomarkers of optic neuritis with those from healthy controls

| Serum biomarkers in healthy controls (previous studies) | | |
| --- | --- | --- |
|  | Serum NfL, pg/mL (IQR) | Serum GFAP, pg/mL (IQR) |
| Aktas et al.^18^ (n = 85) | – | 71.3 (55.6–102.2) |
| Kim et al.^30^ (n = 22) | 10.2 (8.0–14.0) | 98.9 (61.3–142.4) |
| Shahim et al.^47^ (n = 68) | 6.3 (3.6–9.2) | 60.2 (46.4–80.1) |
| Hogel et al.^48^ (n = 13) | 22.9 (11.5–36.6) | 69.1 (42.2–137.0) |
| Serum biomarkers in patients with optic neuritis (the present study) | | |
|  | Serum NfL, pg/mL (IQR) | Serum GFAP, pg/mL (IQR) |
| AQP4-ON | 12.2 (7.0–27.5) | 269.1 (111.7–342.1) |
| MOG-ON | 9.9 (8.2−12.8) | 62.5 (48.8–95.7) |
| DSN-ON | 9.4 (5.9−14.5) | 77.6 (61.0–109.9) |

AQP4-ON, optic neuritis with aquaporin-4 antibody; DSN-ON, double-seronegative optic neuritis; GFAP, glial fibrillary acidic protein; IQR, interquartile range; MOG-ON, optic neuritis with myelin oligodendrocyte glycoprotein antibody; NfL, neurofilament light chain; ON, optic neuritis.
